# Combined Aerobic and Resistance Training Performed under Conditions of Normobaric Hypoxia and Normoxia Has the Same Impact on Metabolic Control in Men with Type 1 Diabetes

**DOI:** 10.3390/ijerph182413058

**Published:** 2021-12-10

**Authors:** Marta Wróbel, Dominika Rokicka, Artur Gołaś, Miłosz Drozd, Alicja Nowowiejska-Wiewióra, Łukasz Pyka, Tomasz Stołtny, Mariusz Gąsior, Krzysztof Strojek

**Affiliations:** 1 Department of Internal Medicine, Diabetology and Cardiometabolic Disorders, Faculty of Medical Sciences Zabrze, Medical University of Silesia, 40-055 Katowice, Poland; dominika.rokicka@poczta.fm (D.R.); kstrojek@sum.edu.pl (K.S.); 2 Department of Sports Training, The Jerzy Kukuczka Academy of Physical Education, 40-065 Katowice, Poland; a.golas@awf.katowice.pl (A.G.); m.drozd@awf.katowice.pl (M.D.); 3 3rd Department of Cardiology, Faculty of Medical Sciences Zabrze, Medical University of Silesia, 40-055 Katowice, Poland; anwiewiora@tlen.pl (A.N.-W.); wookash.p@gmail.com (Ł.P.); mgasior@sum.edu.pl (M.G.); 4 District Hospital of Orthopaedics, Trauma Surgery in Piekary Śląskie, 41-940 Piekary Śląskie, Poland; quattro42@poczta.onet.pl

**Keywords:** aerobic training, resistance training, exercise, type 1 diabetes

## Abstract

(1) Background: The aim was to assess whether combined aerobic and resistance training performed under hypoxic and normoxic conditions had an impact on diabetes control, VO_2_max (maximum oxygen consumption), and echocardiological and anthropometric parameters in men with long-term type 1 diabetes. (2) Methods: Sixteen male participants (mean age: 37 years, mean HbA1c (glycated hemoglobin): 7.0%) were randomly assigned to two groups: training in normoxic conditions or training in conditions of altitude hypoxia. All subjects participated in 60 min combined aerobic and resistance training sessions twice a week for 6 weeks. At baseline and in the 6th week, echocardiography, incremental exercise test, and anthropometric and diabetes control parameters were assessed. (3) Results: After 6 weeks, there was no significant change in HbA1c value in any group. We noted a more stable glycemia profile during training in the hypoxia group (*p* > 0.05). Patients in the hypoxia group required less carbohydrates during training than in the normoxia group. A comparable increase in VO_2_max was observed in both groups (*p* > 0.05). There were no significant differences in cardiological and anthropometric parameters. (4) Conclusions: Combined aerobic and resistance training improved VO_2_max after 6 weeks regardless of the conditions of the experiments. This exercise is safe in terms of glycemic control in patients with well-controlled diabetes.

## 1. Introduction

In addition to proper nutrition, exercise is the cornerstone of behavioral treatment for all types of diabetes. The benefits of aerobic exercise in people with diabetes have been widely proven. This exercise improves insulin sensitivity, leads to weight reduction, and improves vascular reactivity, endothelial function, lipid profile, lung function, cardiorespiratory fitness, and cardiac output. Moderate aerobic exertion reduces overall and cardiovascular mortality [1,2]. Many diabetes societies also permit resistance training to be used in people with diabetes [3]. Resistance training has a positive effect on muscle mass, body composition, bone density, and cardiovascular parameters [4,5]. Both forms of exercise can remodel the heart muscle and affect the function of the left ventricle [6]. In recent years, training under normobaric hypoxia has been used more and more often in sports training programs and in rehabilitation. Hypoxic conditions are produced artificially in a training room, in which the air consists of 15.4% oxygen and 84.7% nitrogen (which imitates the conditions at an altitude of 2500 m above sea level). Training in a hypoxic environment is now also available commercially in some training rooms. Training in hypoxic conditions may contribute to the improvement of exercise capacity in normoxic conditions as a result of adaptive changes being triggered in the body [7]. Hypoxia induces the production of the hypoxia inducible factor (HIF-1), which regulates the expression of over 100 genes, including the gene responsible for the synthesis of erythropoietin. HIF-1 also leads to an increase in angiogenesis in muscle tissue and has a positive effect on energy processes, including glycolysis. Under hypoxic conditions, adaptive changes in the respiratory and cardiac system occur and the density and network of capillaries in the muscles and muscle hypertrophy increase, as do the concentration of myoglobin and the number of mitochondria in cells [8]. Patients with type 1 diabetes have an increased risk of developing complications due to many years of exposure to elevated glucose levels. In addition to classic locations such as kidneys, retina, peripheral nerves, heart, and large vessels, complications may also relate to muscle tissue in the form of muscle atrophy and muscle strength reduction [9,10]. IGF-1 is the main factor controlling muscle growth and the proper functioning of the neuromuscular system. Patients with type 1 diabetes have decreased IGF-1 expression which leads to disturbances in bone metabolism and neuropathy, as well as muscle atrophy and cardiovascular complications. It has been shown that physical exercise performed in conditions of moderate hypoxia (2500 m above sea level) leads to an increase in the concentration of free IGF-1 [11]. Exercise in hypoxia also increases NO production and lowers the concentration of pro-inflammatory TNF-alpha. The vasodilating effect obtained in this way and the reduction of low-grade inflammation may have a positive effect on the condition of blood vessels in diabetic patients [12]. Hypoxia also increases the oxidative activity of mitochondrial enzymes and improves the use of carbohydrates [13]. It has been shown that training under hypoxic conditions improves glucose tolerance and insulin sensitivity in patients with type 2 diabetes and obese persons with metabolic syndrome compared to patients training under normoxic conditions [14,15]. Moreover, in obese patients with type 2 diabetes, sleep under hypoxic conditions imitating an altitude of 2400 m above sea level for 10 nights resulted in a reduction of fasting glucose and an improvement in insulin sensitivity [16]. In high mountain populations, coexistence of increased glucagon levels with low blood glucose levels has been observed, which may indicate a decreased liver sensitivity to glucagon, which results in a decrease in hepatic glucose production [17].

The above-mentioned benefits of training conducted under hypoxic conditions on glucose concentration and muscle tissue in combination with mixed training, which is associated with a more stable course of glycemia (in comparison to aerobic exercise alone), provided the reason to design the following study in type 1 diabetic patients. The aim of the study was to evaluate the effect of combined aerobic and resistance exercise conducted in type 1 diabetic patients in hypoxic conditions on glycemic profile and its stability (with use of an intermittent scanning glucose monitoring system), HbA1c value, frequency and severity of hypoglycemia, VO_2_max, body weight, waist-to-hip ratio (WHR), body composition (tissue and muscle content), insulin requirements, and the need to replenish carbohydrates during exercise in comparison to the same type of effort in normoxic conditions. To date, the impact of combined aerobic and resistance training in hypoxic conditions on the above parameters in patients with type 1 diabetes has not been assessed.

## 2. Material and Methods

### 2.1. Experimental Approach to the Problem

Before starting the training, all patients underwent a preliminary evaluation in order to qualify for inclusion in the study:

- Echocardiographic examination;
- ECG (electrocardiogram) exercise test on a treadmill;
- Ophthalmological evaluation (fundus examination was regarded as valid when performed no longer than a year before training started).

After a positive evaluation by a cardiologist, 16 patients were randomly assigned to one of two groups:

1. Training in normoxic conditions—eight patients;
2. Training in conditions of altitude hypoxia (normobaric)—eight patients.
3. Randomization was carried out in blocks of four people.

### 2.2. Subjects

Sixteen men with type 1 diabetes from the area of Silesia (Poland), with low baseline physical activity (declared that they were usually taking 4000–6000 steps a day) and without advanced complications of diabetes, were included in the study. The study was conducted from October 2019 to August 2020.

Patients were included in the study according to the following inclusion criteria:

- Type 1 diabetic patients aged 30–45 years, diabetes duration at least 10 years, BMI (body mass index) 20–30 kg/m^2^;
- Treated with multiple insulin injections (at least 4/day) or with a personal insulin pump;
- Negative exercise ECG test;
- HbA1c < 8.0%;
- Treatment with intensive functional insulin therapy, counting carbohydrate exchanges (1 carbohydrate exchange = 10 g of carbohydrates), knowledge of how to manage diabetes during physical exercise;
- Advanced operation of the glucose meter and intermittent scanning glucose monitoring system—the FreeStyle Libre system (Abbott Diabetes Care, Alameda, CA, USA).

Exclusion criteria:

- Advanced microvascular complications of diabetes: (pre-proliferative and proliferative retinopathy, state after laser therapy; GFR (glomerular filtration rate) less than 60 mL/min/1.73 m^2^, microalbuminuria);
- Macrovascular complications: cardiovascular disease;
- Overt autonomic neuropathy (including no increase in heart rate with exercise).

The general characteristics of the group are presented in Table 1.

The study protocol was approved by the Bioethics Committee at the Medical University of Silesia in Katowice (PCN/0022/KB1/102/19). ClinicalTrial.Gov number: NCT04450745. Written consent was obtained from all participants of the study after explaining the purpose of the study. All participants who met the inclusion criteria and did not meet the exclusion criteria were subjected to initial clinical examinations, including cardiological, to assess their health status before starting training.

**Table 1:** General group characteristics: Baseline assessment and in 6th week in both groups.

Variable	Training in Hypoxia at Baseline	Training in Hypoxia in 6th Week	Training in Normoxia at Baseline	Training in Normoxia in 6th Week	*p*
Age (years)	38.8 ± 4.32		37.5 ± 5.32		ns
Diabetes duration (years)	21 ± 9		16 ± 5		ns
Height (cm)	181 ± 9.0		182.5 ± 4.6		ns
Weight (kg)	89.7 ± 16.5	89.6 ± 16.7	94.1 ± 6.4	93.6 ± 6.3	ns
BFM (kg)	22 ± 4.6	21.8 ± 5.12	22.8 ± 6.6	22.6 ± 7.6	ns
SMM (kg)	38 ± 7.5	38 + 7.6	40.4 ± 2.4	40.3 ± 2.6	ns
BMI (kg/m^2^)	27.1 ± 2.7	27.0 ± 2.8	28.2 ± 1.5	28.1 ± 1.5	ns
PBF (%)	24.5 ± 2.7	24.4 ± 3.3	24.05 ± 5.5	23.8 ± 6.5	ns
WHR	0.964 ± 0.06	0.962 ± 0.06	0.985 ± 0.07	0.98 ± 0.08	ns
Circumference of abdomen (cm)	100 ± 11	99 ± 11	103 ± 9	103 ± 10	ns
Circumference of hip (cm)	103 ± 6	103 ± 6	105 ± 2	105 ±2	ns
HbA1c (%)	6.93 ± 0.7	6.87 ± 0.7	7.0 ± 0.6	6.96 ± 0.7	ns
HbA1c (mmol/mol)	52 ± 8	51.6 ± 8	53 ± 7	52.6 ± 7	ns
Creatinine (μmol/L)	76 ± 14	73 ± 11	72 ± 6	69 ± 6	ns
ALT (U/L)	21 ± 6	24 ± 7	23 ± 11	24 ± 12	ns
AST (U/L)	26 ± 10	26 ± 12	23 ± 9	23 ± 7	ns
EQ-5D VAS (points)	82 ± 17	86 ± 9	78 ± 13	82 ± 13	ns
Short-acting insulin (U/24 h)	36 ± 13	36 ± 11	41 ± 16	30 ± 11 *	ns
Long-acting insulin (U/24 h)	24 ± 8	23 ± 9	29 ± 10	28 ± 11	ns
Threshold for hypo signs (mg/dl)	58 ± 9	64 ± 8	58 ± 11	60 ± 10	ns
Self-reported hypoglycemia (episodes/week)	2.5 ± 1.8	3.2 ± 2.4	3.6 ± 3.2	4.5 ± 2.8	ns

Values are means ± standard deviation. No significant differences were observed between groups in subjects’ characteristics at entry into the study. * *p* < 0.05 baseline vs. 6th week within the group. Remaining comparisons were not significant. BFM: body fat mass, SMM: skeletal muscle mass, BMI: body mass index, PBF: percent body fat, WHR: waist-to-hip ratio, EQ-5D VAS: quality of life assessment.

### 2.3. Instruments and Procedures

Before starting the training, the participants were subjected to an incremental exercise test and muscle strength test at the Department of Theory and Practice of Sport of the Academy of Physical Education in Katowice.

#### 2.3.1. Incremental Exercise Test

An exercise test of increasing intensity was performed by all patients enrolled in the study. Incremental exercise test is a useful tool for determining an individual’s physical fitness level and adaptation to a training program. The test was carried out on a Pulsar treadmill (HP Cosmos, Nussdorf-Traunstein, Germany). The test protocol included an output load of 6 km/h followed by an incremental load increase of 1.5% every 3 min. The test was continued until the subject’s refusal. During the test, continuous recording of heart rate (HR), pulmonary minute ventilation (VE), respiratory rate (BF), oxygen uptake (VO_2_), and exhaled carbon dioxide (VCO_2_) was carried out using the MetaLyzer 3B-2R rapid gas analyzer (Cortex, Leipzig, Germany).

#### 2.3.2. Muscle Strength Test

The maximum load of 1RM was determined during two resistance exercises: pressing the bar while lying on a flat bench (barbell bench press) and lifting the bar forward (barbell front raise). Lifting the bar forward is a basic exercise involving the deltoid muscles. Front barbell raises also work the serratus anterior, along with the upper and lower trapezius, clavicular part of the pectoralis major, and biceps brachii. Before starting the study, the subjects performed a 15 min warm-up, including 10 min of work on the M3 Total Body Trainer ergometer, and several resistance exercises involving the upper and lower limbs. The 1RM value was determined for each subject according to the procedure of Baechle et al. 2008 [18]. The formula was used to determine the value of 1 RM:1 RM = load × (1 + 0.033x the number of repetitions performed).

#### 2.3.3. Training Sessions

After the baseline testing, the patients began their training program. Training sessions were held at the Hypoxia Laboratory and the Muscle Strength and Power Laboratory of the Academy of Physical Education in Katowice twice a week for 60 min for a period of 6 weeks (on Monday/Thursday or Tuesday/Friday). The subjects could not perform additional muscle strength training. All patients assigned to both groups followed the same training program, with individually selected loads in terms of both performance parameters and external loads in muscle strength training. Each of the subjects was trained in the correct technique of performing strength exercises. Each training unit was preceded by a 15 min standardized warm-up including a general warm-up of approximately 5 min using a hand cycle ergometer (heart rate of around 130 bpm), followed by a general upper-body warm-up of 10 trunk rotations and trunk side-bends on each side, 10 internal and external rotary movements of the shoulders, and 10 push-ups. During the specific warm-up, the participants performed 15 repetitions at 20% of their estimated 1RM followed by 10 repetitions at 40% 1RM.

In both groups, the study protocol was a combination of aerobic and resistance training. Aerobic training was interspersed with resistance training involving training with an accent focused on eccentric work (pace 3010—3 s eccentric work, 1 s concentric work). For the aerobic part of training, the range of aerobic processes covered the value of 50–70% HRmax (maximum heart rate) (Oxygen Zone—Low Intensity). Each training session consisted of 10 sets of 1 min of aerobic treadmill training and 10 repetitions of the resistance exercise with a load of 50% 1RM. For the resistance part of the training, two exercises were selected—barbell bench press and barbell front raise (the same exercises were used in the strength test). The training periodization included changes in intensity through changes in running speed, external load, and the pause time between efforts. Once a week, the load was increased by 2.5 kg in order to properly adapt to the supercompensation processes. When the participant could not complete the planned number of repetitions, the load remained at the previous level. For aerobic training, adaptation included a transition from 50% to 75% HRmax (an increase of about 5% every week). 

To perform training sessions in hypoxia, the proper conditions were created in a hypoxia chamber, in which a fractional concentration of inspired oxygen (FiO_2_) was 15.4% with normobaria (atmospheric pressure of 990 hPa). Created conditions were equivalent to that at an altitude of 2500 m above sea level. The hypoxic environment was produced by the Altitude Trainer Hypoxico System (HYP-123 Hypoxic Generator, LOWOXYGEN Technology GmbH, Berlin, Germany). Normoxic conditions were standard indoor air with a FiO_2_ of 20.9% and atmospheric pressure of 990 hPa

#### 2.3.4. Baseline Assessment

In addition to the preliminary studies discussed above, the following were performed:

HbA1c, morphology, AST (aspartate transaminase), ALT (alanine transaminase), creatinine, urine albumin (if not checked in the last year), lipid profile, body weight, BMI, waist and hip circumference, body composition (body mass and composition were evaluated in the morning (7:00–8:00 a.m.) after an overnight fast using the electrical impedance method (Inbody 720, Biospace Co., Tokyo, Japan); food and liquid intake was monitored the night before these measurements), quality of life (all participants completed the EuroQol-5D VAS worksheet, which assesses wellbeing and quality of life using a scoring system of 0–100 points, where 0 means the worst and 100 the best imaginable health state [19]), baseline insulin requirement (1 week before training started), and baseline glucose control based on data read from FreeStyle Libre (intermittent scanning glucose monitoring system, Abbott Diabetes Care, Alameda, CA, USA) via Libreview cloud-based system, with particular emphasis on the following parameters:

1. TIR (time in range; %): the percentage of time that a person spends with their blood glucose levels in the target range: 70–180 mg/dl;
2. TBR (time below range): the percentage of readings and time per day below the target glucose range: level 1: (glycemia < 70 mg/dl (range: 54–69 mg/dl)), level 2: (glycemia < 54 mg/dl);
3. TAR (time above range): the percentage of readings and time per day above the target glucose range (glycemia > 180 and <250 mg/dl);
4. CV (coefficient of variation): [(SD of glucose)/(mean glucose)], value below 36% appears to be a suitable threshold to distinguish between stable and unstable glycemia.

All participants underwent initial training on how to handle physical exertion (adjusting insulin doses, estimating carbohydrate exchanges) and learning how to use the FreeStyle Libre system. Glycemia was monitored using the FreeStyle Libre system in all patients. Obligatory additional readings of blood glucose were required on the training day, i.e., 15 min before training, after 30 min of training, and immediately after training completion.

The principles of dietary management in the peritraining period were discussed, giving particular attention to the composition and timing of the meals immediately before and after the training (patients also received these instructions in writing).

#### 2.3.5. Assessment during the Study Period

- Any type of nutrition on days without training;
- On the day of training, the fixed composition of the meal before exercise (each participant was obliged to eat a carbohydrate–protein meal with a fixed carbohydrate content. This meal had to contain a minimum of 4–5 complex carbohydrate exchanges (40–50 grams of carbohydrates) plus a protein content equivalent to 100 g of poultry meat), a meal eaten no later than 2 h before the exercise;
- Target blood glucose concentration before exercise 140–180 mg/dl;
- Completion of diaries by patients, in which they wrote down the consumed amount of carbohydrate exchanges immediately before, during, and immediately after exercise to keep glycemia at a safe level, and their doses of insulin;
- Reading data from FreeStyle Libre system.

#### 2.3.6. Assessment in 6th Week of the Study

- Incremental exercise test in both groups at Academy of Physical Education in Katowice;
- HbA1c, morphology, AST, ALT, creatinine, body weight, BMI, waist and hip circumference, body composition, quality of life according to the EQ-Worksheet questionnaire, insulin requirement (average dose from 7 days before the end of the study), analysis of hypoglycemia, and the amount of carbohydrates consumed per training session (based on data from the diary);
- Reading data from FreeStyle Libre system.

### 2.4. Statistical Analysis

In order to characterize the structure of the studied variables, the means and standard deviations or medians and interquartile ranges were calculated. The Shapiro–Wilk test was used to verify the normality of the distributions of the analyzed variables. In order to verify the significance of differences before and after the training, significance-of-differences tests for dependent samples were used. If the variables had normal distributions, Student’s *t*-test for dependent samples was used. After finding extreme asymmetries, the Wilcoxon pairwise test was used. All analyses were performed using Statistica v.13. For all analyses, the level of significance was 0.05.

## 3. Results

The baseline characteristics of the participants are presented in Table 1.

### 3.1. Diabetes Control, Hypoglycemia, and Insulin Requirements

The studied groups did not differ at baseline in terms of HbA1c: 6.9% (52 mmol/mol) in the hypoxia group vs. 7.0% (53 mmol/mol) in the normoxia group, *p* = 0.6. There was no statistically significant difference in HbA1c between groups after 6 weeks of training.

**Figure 1:**
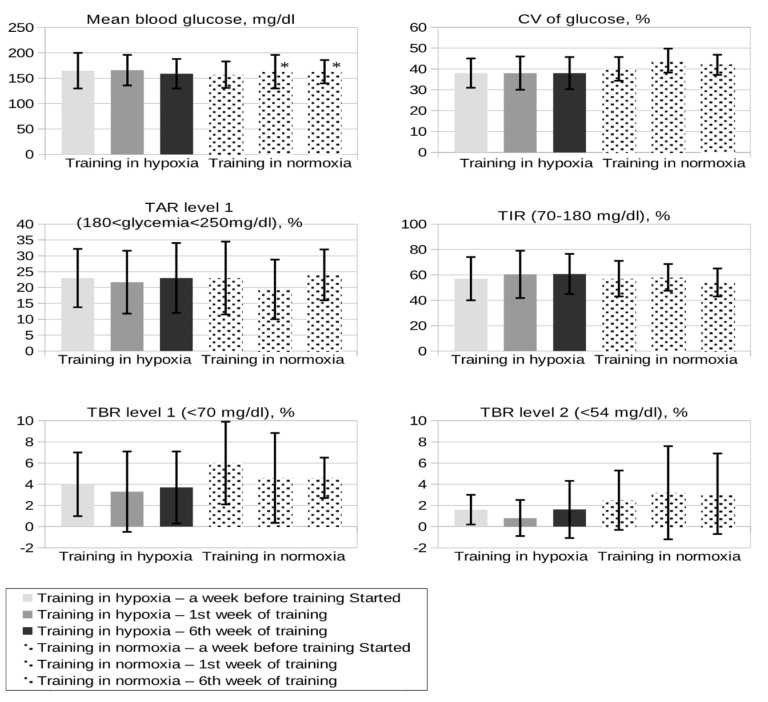
Glycemia according to FreeStyle Libre: a week before training, in the 1st week of the training program, and in the 6th week of the training program in both groups. Training in hypoxia is shown in plain bars and that in normoxia with dotted bars. Values are means ± standard deviation. Comparisons were not significant. * *p* < 0.05 baseline vs. 1st and 6th week within the group. CV (coefficient of variation) of glucose = [(SD of glucose)/(mean glucose)]; <36% appears to be a suitable threshold to distinguish between stable and unstable glycemia. TIR (time in range; %): the percentage of time that a person spends with their blood glucose levels in the target range of 70–180 mg/dl. TBR: percentage of readings and time per day below target glucose range: level 1 (glycemia < 70 mg/dl), level 2 (glycemia < 54 mg/dl). TAR: percentage of readings and time per day above target glucose range (glycemia > 180 < 250 mg/dl).

We did not observe a statistically significant difference in the mean glucose concentration between the groups, either in the week before training started or during the first and last weeks of training (Figure 1). There was a slight significant increase in mean blood glucose value during the 1st and 6th weeks of training in comparison to the week before training started in the normoxia group (Figure 1). Regarding other data from FreeStyle Libre, TIR (time in range; the percentage of time that a person spends with their blood glucose levels in the target range of 70–180 mg/dl) was similar at baseline for both groups, but we observed a nonsignificant slight increase in TIR in the hypoxia group in the 1st and 6th weeks of training (Figure 1). The coefficient of variation (CV = [(SD of glucose)/(mean glucose)]) was also slightly lower and stable during the whole training period in the hypoxia group in comparison to the normoxia group (mean = 38% in the week before training started and within the 1st and 6th week of training vs. 40% baseline and 44% in the 1st and 42% in the 6th week in the normoxia group); the difference between the groups was not significant. When we summarized results from the last six training sessions, we found that the mean glucose value in the middle of a training session (30th minute) was lower in the normoxia group vs. hypoxia (115 mg/dl vs. 157 mg/dl, *p* = 0.06). A similar tendency was seen in glycemia directly after training and regarding fasting glycemia the next morning (Table 2) in the normoxia group vs. the hypoxia group. Those differences were not statistically significant.

We did not find significant differences regarding hypoglycemia between groups. Baseline (assessed in the week before training started) time below range (TBR) level 1 (percentage of readings and time spent in glycemia range: 54–69 mg/dl) was higher in the normoxia group (TBR of 6%) in comparison to the hypoxia group (TBR of 4%). At the end of the study (6th week), a slight insignificant downward trend was seen in both groups, but values in the normoxia group did not achieve the desired goal of TBR below 4%, in contrast to the hypoxia group (*p* > 0.05). Regarding TBR level 2 (percentage of readings and time spent in glycemia below 54 mg/dL) we noted a lower tendency to hypoglycemia in the hypoxia group during the whole study period, with achievement of a desired value below 1% in the first week of training (Figure 1). Moreover, the level of carbohydrate consumption directly before training and within a training session to prevent hypoglycemia was higher in the normoxia group, but the difference between the groups was not statistically significant (*p* > 0.05; Table 2). There were no significant changes in threshold for hypoglycemia signs (data self-reported by participants) and no differences in self-reported hypoglycemia episodes weekly, but a slight upward trend was seen in the normoxia group (Table 1). Throughout the study, no severe hypoglycemia occurred in any of the participants.

In the normoxia training group, the daily short-acting insulin requirement decreased significantly after 6 weeks compared to baseline (41 U vs. 30 U/day, *p* < 0.05, Table 1). However, we did not observe a significant difference in this parameter between the groups. We also observed a significantly higher dose reduction of short-acting insulin before the last meal before the training session in the normoxia group (25% vs. 10% in hypoxia, *p* < 0.05 Table 2). 

### 3.2. Anthropometric, Biochemical, and Cardiac Parameters

We did not observe statistically significant differences between baseline and end values between groups in terms of body weight; BMI; WHR; fat mass; muscle mass (body composition); echocardiography; creatinine, alanine, and aspartate transaminases; or complete blood counts (data not shown).

Data are presented in Table 1 and Table 3.

### 3.3. Incremental Exercise Test

The statistical analysis did not show any significant influence of training in either hypoxia or normoxia on selected cardiorespiratory variables (Table 4). However, there was a nonsignificant upward trend in the final load (VO_2_max) in both groups after 6 weeks of training, with visible change in the maximum heart rate.

**Table 2:** Characteristics of the peritraining period (mean values from last 6 training sessions): comparison between the groups.

Variable	Training in Hypoxia	Training in Normoxia	*p*-Value
Dose reduction of short-acting insulin premeal before training (%)	10 ± 2.8 *	25 ± 14	0.004
Mean glucose 15 min before training (mg/dl)	169 ± 36	141 ± 40	0.2
Mean glucose in 30 min of training (mg/dl)	157 ± 38	115 ± 40	0.06
Mean glucose at the end of training (mg/dl)	132 ± 36	114 ± 33	0.4
Extra carbs during training (grams)	10 ± 6	18 ± 30	0.7
Mean fasting glycemia in the next morning (mg/dl)	128 ± 62	102 ± 36	0.8

Values are means ± standard deviation. * *p* < 0.05 baseline vs. 6 week within the group.

**Table 3:** Echocardiography at baseline and after 6 weeks: left ventricular ejection fraction and right ventricle parameters.

Variable	Training in Hypoxia at Baseline	Training in Hypoxia in 6th Week	Training in Normoxia at Baseline	Training in Normoxia in 6th Week
Ejection fraction (%) biplane	59.5 ± 3	59.7 ± 4	63.6 ± 4	62 ± 6
RVMODE (mm)	28.75 ± 2.12	29.2 ± 2.6	28.8 ± 2	28.8 ± 2.5
RVLV4CH (mm)	34.3 ± 2.7	34.3 ± 2.7	34 ± 2.5	34.6 ± 2.2
RVSP (mmHg)	15.4 ± 5	17.3 ± 2	17.8 ± 10	19.4 ± 8.5

RVMODE: right ventricular m mode dimension, RVLV4CH: right to left ventricular diameter ratio in the four-chamber view, RVSP: right ventricular systolic pressure.

**Table 4:** Incremental exercise test at baseline and after 6 weeks.

Variable	Training in Hypoxia at Baseline	Training in Hypoxia in 6th Week	Training in Normoxia at Baseline	Training in Normoxia in 6th Week
HR max (bpm)	172 ± 14	168 ± 12	178 ± 15	171 ± 15
RER	1.3 ± 0.07	1.2 ± 0.07	1.2 ± 0.11	1.18 ± 0.13
V′O_2_/kg (mL/min/kg)	36.6 ± 4.7	42.0 ± 6.0	38.5 ± 4.5	42.4 ± 4.5
V′O_2_/HR (mL)	19.5 ± 3.8	21.05 ± 4.5	22 ± 3.1	22.7 ± 3.9
VT	3.3 ± 0.88	3.2 ± 0.6	3.1 ± 0.35	3.1 ± 0.4

Values are means ± standard deviation. Comparisons were not significant. HR: heart rate, bpm: beats per minute, RER: respiratory exchange ratio, V′O_2_: oxygen consumption, BPM: beats per minute, VT: tidal volume.

### 3.4. Quality of Life

We observed an upward trend in the quality of life in both groups at the end of the study as compared to baseline, with no differences between the groups (*p* = 0.34) (Table 1).

### 3.5. Safety

No side effects were recorded during the study.

## 4. Discussion

The aim of the study was to evaluate the effect of combined aerobic and resistance exercise conducted in hypoxic in comparison to normoxic conditions on diabetes control, VO_2_max, and anthropometric, echocardiographic, and laboratory parameters in patients with long-term type 1 diabetes, initially inactive. Our results show that supervised training of combined aerobic and resistance exercise, conducted in both normoxia and normobaric hypoxia over a period of 6 weeks, with the frequency of two 60 min sessions per week, equally affected the control of diabetes (HbA1c, time in range, mean glycemia, CV, frequency of hypoglycemia), VO_2_max, and echocardiographic, laboratory, and anthropometric parameters. Our work is the first study to assess the effect of combined training on the above parameters in both normoxic and hypoxic conditions in patients with type 1 diabetes.

The baseline HbA1c values in both groups (6.9% and 7.0%) indicated very good metabolic control of diabetes in the study participants. Therefore, we did not expect a reduction of this parameter after a 6 week training period. Greater reductions in HbA1c could have been obtained if patients with an elevated value of glycated hemoglobin were included in the study. When using continuous glucose monitoring (CGM) or intermittent scanning CGM systems (FreeStyle Libre, Abbott Diabetes Care, Alameda, CA, USA), we used parameters other than HbA1c to assess the degree of diabetes control. A TIR of 70% meant very good alignment, convergent with HbA1c of less than 7.0%. The participants of our study had TIR values lower than desired at baseline, but in the hypoxia group we observed a favorable upward trend in TIR compared to the normoxia group (60% vs. 54% in the 6th week of the study, *p* > 0.05) and lower glucose fluctuations (coefficient of variation (CV) of 38% vs. 42–44% in the normoxia group).

In the hypoxia group, patients reduced their dose of insulin with a meal before training to a lesser extent, which did not affect the incidence of hypoglycemia in this group. Glycemia during training was more stable in the hypoxia group, and patients required lower amounts of carbohydrate supplementation during training to prevent hypoglycemia than those training under normoxic conditions (Figure 1). The mean fasting glycemia the next day was also slightly higher in the hypoxia group compared to the normoxia group (128 mg/dl vs. 102 mg/dl, respectively). Overall, training under moderate hypoxia appears to be associated with a more stable course of glycemia. Literature data on the effect of moderate hypoxia on blood glucose are scarce. Nevertheless, it has been shown that hypoxia promotes lowering of blood glucose levels through the stimulating effect of HIF-1 on glycolysis and as a result of the increased expression of GLUT4 glucose transporters in muscle tissue independently of muscle work [13]. In one of the few studies in patients with type 1 diabetes, a single 40 min moderate-intensity exercise bout (50% of lactate threshold) on a cycloergometer in hypoxia was associated with a reduction in glycemia immediately after training and within 24 h of recovery compared to the same effort under normoxic conditions [11]. Regardless of the conditions in which the training took place, it was a moderate effort that usually led to lowering of glycemia in diabetic patients. Another study analyzed the effect of two types of exercise in patients with type 1 diabetes: 40 min of continuous exercise of moderate intensity (50% of lactate threshold and 66% of maximal heart rate) and intermittent exercise of high intensity (120% of lactate threshold and 90% of maximal heart rate) with a duration of 4 × 5 min with a 5 min rest after each bout of exercise, performed in both hypoxia and normoxia. For both types of training, no statistically significant differences between the groups (hypoxia vs. normoxia) were found [20]. In type 1 diabetes, the effects of exercise on glucose levels are the result of many factors, including pre-exercise glucose values, injection site, amount of insulin administered, composition and size of the pre-exercise meal, and duration and intensity of exercise. Exercise in patients treated with insulin is associated with an increased risk of hypoglycemia because the concentration of exogenous insulin is not able to drop immediately, as is the case at the beginning of exercise in healthy people with functional beta cells of the pancreas. In addition, better blood supply to the subcutaneous fat during physical exercise accelerates the absorption of insulin from the injection site and thus exposes the patient to faster hypoglycemic effects [21]. The participants of our study were obliged not to start training earlier than 1.5–2 h after the administration of the last dose of preprandial insulin, so that the training time did not fall within the maximum effect of the hormone. It is known that physical exercise, especially aerobic, lowers glycemia after just a few minutes. Shrinking of skeletal muscles increases the sensitivity of muscle cells to insulin due to the translocation of GLUT-4 glucose transporters into the cell membrane, which permits diffusion of circulating glucose into cells in an insulin-independent manner [22]. On the other hand, resistance exercise, especially short-term and intense, may lead to post-workout hyperglycemia [23] as a result of, for example, adrenaline stimulating hepatic gluconeogenesis. Our study participants performed combined aerobic and resistance training. This type of training has a beneficial effect on the course of glycemia in patients with type 1 diabetes [23,24], as we have also shown in our work and discussed above. In a recent study conducted in type 1 diabetic patients, it was found that fewer episodes of hypoglycemia occurred within 24 h after 40 min of continuous, moderate-intensity exercise under hypoxic conditions, which is consistent with our results [20]. On the other hand, high-intensity training was associated with a greater risk of hypoglycemia the next day, which could result from a deficiency of glycogen at a later stage after it was used as fuel during resistance training [25]. Regarding hypoglycemia, our results, despite the lack of difference between the groups, may suggest that combined aerobic and resistance training conducted under hypoxic conditions may be a safer option in relation to the risk of exercise-induced hypoglycemia. We also found that such training in hypoxia required lower carbohydrate consumption within a training session and there was lower need for short-acting insulin dose reduction to prevent hypoglycemia, in comparison to normoxia (*p* > 0.05). 

According to the available literature, the initial VO_2_max of the participants in our study can be considered good and appropriate for their age. A maximal oxygen uptake (VO_2_max) of at least 37 mL/kg/min shows good exercise tolerance [18,26]. The participants of our study showed improved VO_2_max after 6 weeks of training, which suggests a beneficial effect of the exercise intervention used regardless of whether the training took place in conditions of hypoxia or normoxia. However, the difference observed by us both within the group (VO_2_max at the end of the study in relation to the baseline values) and between the groups did not reach statistical significance. This may indicate good training periodization and appropriately selected external loads sufficient to induce adaptive changes in the patients regardless of the conditions of the experiments. In studies evaluating the effect of mixed training under hypoxic conditions in healthy athletes (without diabetes) on VO_2_max, increases of 8–10% were obtained after a 5–6-week training period [27]. In the latest literature related to sports training, anaerobic training plays a very important role in improving VO_2_max. 

It is surprising that there were no changes in the body composition of our participants, especially in the hypoxic group, because training in such conditions leads to greater muscle hypertrophy due to, among other factors, increased HIF-1 production and increased IGF-1 expression. Perhaps this was a result of disturbed muscle structure as a result of many years of diabetes (mean disease duration of 21 years in the hypoxia group and 16.5 years in the normoxia group). A longer duration of the study might have allowed observation of possible changes in body composition.

The lack of significant changes in the echocardiographic examination, especially in the area of the right ventricle and right atrium of the heart, suggests that a 6 week period of combined aerobic and resistance training performed in both hypoxic and normoxic conditions is safe for patients with type 1 diabetes. 

## 5. Conclusions

Despite the lack of a statistically significant improvement in glycemic control expressed as lowering of HbA1c value, mean glycemia, and increasing time spent in the desired glycemic range (TIR), we observed that combined aerobic and resistance training performed under hypoxic conditions ensured a more stable course of glycemia both during and after training sessions. The patients exercising under hypoxia showed a lower tendency to hypoglycemia, and they had lower glucose fluctuations with the increasing TIR, which was not observed in the group training in normoxia. In both the hypoxia and normoxia groups, a comparable increase in VO_2_max was obtained, which proves that the form of combined aerobic and resistance training we used had a beneficial effect on the cardiovascular system.

### Limitations

The small size of the group is the main limitation of our study. A longer period of observation is also necessary to assess whether the type of training performed by patients, especially in hypoxic conditions, has a clear impact on the control of diabetes, including the stable course of glycemia. The second aspect is the necessity of echocardiographic evaluation of participants after a longer period of training in order to detect the presence of possible adaptive changes in the myocardium in response to the time spent in hypoxia and exercise performed in these conditions. A third aspect is the need for fundus evaluation due to the greater tendency to synthesize VEGF under hypoxic conditions.

## Data Availability

Data available on request due to restrictions e.g., privacy or ethical. The data presented in this study are available on request from the corresponding author.
